# Targeted DNA demethylation of the ZNF334 promoter inhibits colorectal cancer growth

**DOI:** 10.1038/s41419-023-05743-x

**Published:** 2023-03-25

**Authors:** Bin Yang, Haiyu Tang, Nan Wang, Jian Gu, Qin Wang

**Affiliations:** 1 School of Pharmacy, Southwest Minzu University, Chengdu, 610225 Sichuan China; 2 BMI Center for Biomass Materials and Nanointerfaces, College of Biomass Science and Engineering, Sichuan University, Chengdu, 610065 Sichuan China

**Keywords:** Targeted therapies, Apoptosis

## Abstract

Colorectal cancer (CRC) is a leading cause of cancer deaths worldwide. Aberrant regulation of DNA methylation in promoters of tumor suppressor genes or proto-oncogenes is one of the fundamental processes driving the initiation and progression of CRC. Zinc-finger proteins (ZNFs) are one of the most abundant groups of proteins and function in many important biological processes related to tumorigenesis. Herein, we detected abnormal hypermethylation of the ZNF334 gene in CRC tissues compared with normal tissues, and this modification downregulated the expression of ZNF334. Furthermore, ten-eleven translocation 1 (TET1) was identified to be involved in regulating the methylation level of ZNF334. Next, a dCas9-multiGCN4/scFv-TET1CD-sgZNF334-targeted demethylation system was constructed to reverse the expression of ZNF334 through sgRNA targeting the ZNF334 promoter. Both in vitro and in vivo experiments demonstrated the targeted demethylation system upregulated ZNF334 expression and inhibited CRC growth. Collectively, targeted DNA demethylation of the ZNF334 promoter sheds light on the precise treatment of CRC.

## Introduction

Colorectal cancer (CRC) is the third most common malignant neoplasm and the second leading cause of cancer-related death worldwide [1, 2]. Its incidence rate is increasing rapidly in many Asian countries, including China [3]. Besides the aging population and the fattiest diet of high-income countries, adverse risk factors like obesity, physical inactivity, and smoking also increase the risk of CRC [4, 5]. At present, the treatment of CRC mainly includes chemotherapy, surgery, and targeted therapy [6, 7]. When diagnosed at early stages, surgery may be an effective way to control the growth of CRC, but patients are often diagnosed in a later-stage, resulting in a poor prognosis of treatment [6, 8–10]. Furthermore, targeted therapy and cytotoxic chemotherapy have shown promising efficacy in CRC [11, 12]. However, drug resistance, recurrence, and metastasis after treatment still seriously affect the survival of patients. Therefore, it is a great significance task for cancer research to search novel biomarker of molecular targeted therapy of CRC.

DNA methylation is one of the epigenetic modifications that plays an important physiological role in gene transcriptional regulation and developmental processes [13]. Aberrant hypermethylation of cytosine‐phosphoric‐guanine (CpG) islands in the promoter of tumor suppressor genes can lead to transcriptional silencing, thereby promoting the occurrence and development of cancer [14]. Thus, the downregulation of DNA methylation of tumor suppressor genes is a promising approach for cancer therapy [15–17]. In the past few years, biochemical and structural studies have revealed how ten-eleven translocation (TET) mediates the demethylation of active DNA. To achieve a more precise DNA demethylation, the CRISPR–Cas9 has been applied for precise epigenome modification through linkage to epigenome-regulating reagents. A CRISPR-Cas9 system is mentioned consisting of the dead (d) Cas9 fused to the catalytic domain of TET1 (TET1CD). The system has been shown to efficiently regulate the methylation modification of target sites without altering the DNA sequence, and then perform gene regulation.

The ZNFs are one of the largest families of transcription factors and function in many important biological processes related to tumorigenesis [18–20]. ZNFs containing KRAB-ZFPs account for about a third of the 800 different ZNFs [21]. Most members of the ZNFs family play important roles in regulating cell differentiation, cell proliferation, apoptosis, tumor transformation, and anticancer defense [18, 20, 22–24]. ZNF334, a newly identified member of ZNFs, was initially identified as a molecular marker involved in rheumatoid arthritis (RA) and was extremely reduced in CD4 lymphocytes of RA [25, 26]. In addition, it has been found that ZNF334 expression is downregulated in malignant tissues, and its expression is inversely correlated with clinical outcome of patients with various cancers, including hepatocellular carcinoma (HCC) [27, 28] and triple-negative breast cancer (TNBC) [19]. However, the role of ZNF334 in CRC remains unclear.

In this study, we found that ZNF334 downregulation in CRC was DNA methylation-dependent. In addition, we proved that the dCas9-multiGCN4/scFv-TET1CD-sgZNF334 system can target DNA demethylation at specific sites to reverse the expression level of ZNF334, and verified the specificity of this system. Subsequently, both in vitro and in vivo experiments demonstrated the targeted upregulation of ZNF334 expression could inhibit CRC growth. Our results would provide reliable evidence to define the mechanism by which ZNF334 downregulation and its role in CRC. This study provides a new target for early diagnosis and sheds light on the precise treatment of CRC.

## Results

### Promoter methylation and expression of ZNF334 in CRC

**Fig. 1:**
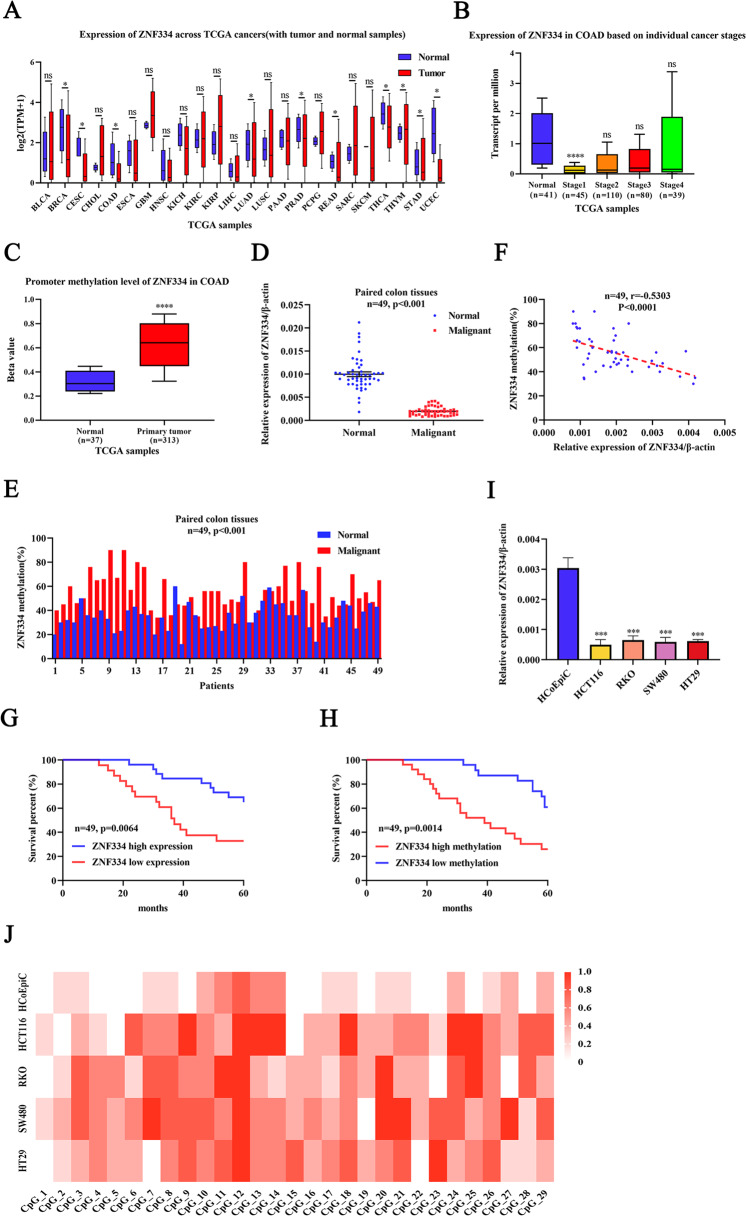
ZNF334 downregulation is modulated by DNA methylation in CRC. **A** Analysis of the ZNF334 expression between tumor and normal tissues from the TCGA database (*p < 0.05). **B** Expression of ZNF334 in individual cancer stages of CRC tissues compared to normal tissues from the TCGA database (****p < 0.0001). **C** Analysis of ZNF334 promoter methylation in CRC tissues compared to normal tissues from the TCGA database (****p < 0.0001). **D** qRT-PCR analysis of ZNF334 expression in 49 patients of CRC and corresponding normal tissues (*n* = 49, *p* < 0.001). **E** ZNF334 promoter methylation status in paired CRC tissues of 49 patients and methylation of ZNF334 was determined by pyrosequencing (*n* = 49, *p* < 0.001). **F** A negative correlation between ZNF334 mRNA expression and promoter methylation in colorectum tissues (Pearson correlation, *p* < 0.0001, *r* = −0.5303). **G** Kaplan–Meier curve showing the overall survival of CRC patients (in percentage), stratified by ZNF334 expression (high expression tumors and low expression tumors; *n* = 49; *p* = 0.0064; long-rank test). **H** Kaplan–Meier curve showing the overall survival of CRC patients (in percentage), stratified by ZNF334 methylation (high methylation tumors and low methylation tumors; *n* = 49; *p* = 0.0014; long-rank test). **I** ZNF334 expression in CRC cells (HCT116, RKO, SW480, and HT29) compared with normal colorectal epithelial cells HCoEpiC detected by qRT-PCR (*n* = 3, ***p < 0.001, compared with the HCoEpiC group). **J** Genomic DNAs extracted from HCoEpiC and CRC cells (HCT116, RKO, SW480, and HT29) were treated with bisulfite and then subjected to methylation-specific PCR, 29 CpG sites were hypermethylated in CRC cells and hypomethylated in HCoEpiC cells.

In this study, to investigate the characteristics of ZNF334 in CRC, we performed an online analysis based on The Cancer Genome Atlas (TCGA) database. Firstly, we compared the expression of ZNF334 between 24 tumor and normal tissues using Pan-cancer view profiling datasets collected in the TCGA project. The results showed that ZNF334 expression was decreased in most tumor tissues compared with normal tissues, and there was a significant difference in the downregulation of ZNF334 expression in CRC tissues (*p* < 0.05, Fig. 1A). We further analyzed the expression of ZNF334 in CRC based on individual cancer stages. The analysis showed that compared with normal tissues, the expression of ZNF334 was significantly downregulated in stage 1 CRC (*p* < 0.0001), while no significant differences were found in stage 2, 3 and 4 CRC (*p* > 0.05) (Fig. 1B). Meanwhile, the methylation degree of the ZNF334 gene promoter in human CRC tissues was significantly higher than that in distal tissues (*p* < 0.0001, Fig. 1C). To further investigate the expression level and promoter methylation status of ZNF334 in CRC, we collected 93 CRC tissues containing clinical stage and prognosis, 49 of which contained paired adjacent normal tissues. Quantitative real-time PCR for ZNF334 using tissues gained from our hospital also demonstrated that ZNF334 was downregulated in 97.96% of human CRC tissues, compared with levels in adjacent normal tissues (Fig. 1D, Normal: 0.00998 ± 0.00051 vs. Malignant: 0.00195 ± 0.00013, *p* < 0.001). A higher methylation percentage was found in malignant tissues (Fig. 1E, Normal: 35.24 ± 1.60% vs Malignant: 55.84 ± 2.16%, *p* < 0.0001). In fact, the mean percentage of CpG methylation in ZNF334 was negatively correlated with its expression level (Pearson’s, r = −0.5303, *p* < 0.0001; Paired colorectum tissues, 49 samples, Fig. 1F), suggesting that DNA methylation represses ZNF334 transcription in human colorectum tumors. Next, the expression and methylation of ZNF334 were divided as “high” and “low” according to the median value. The results showed that the expression of ZNF334 mRNA was a positive prognostic indicator for patients with CRC (*n* = 49, *p* = 0.0064, Fig. 1G). Meanwhile, ZNF334 methylation has a predictive role in CRC patients (*n* = 49, *p* = 0.0014, Fig. 1H). The detailed clinicopathological characteristics of the patients are listed in Supplementary Table 1. In addition, the expression of ZNF334 at the mRNA level in the HCoEpiC and CRC cells (HCT116, RKO, SW480, and HT29) was examined. We observed lower ZNF334 mRNA expression levels in CRC cells than in HCoEpiC (Fig. 1I). Genomic DNA extracted from HCoEpiC and CRC cells was treated with bisulfite and then subjected to bisulfite sequencing PCR (BSP) (Supplementary Table 2). According to BSP sequencing, 29 CpG sites were hypermethylated in CRC cells and hypomethylated in HCoEpiC cells (Fig. 1J). These results suggested that ZNF334 downregulation in human colorectum tumor tissues is DNA methylation-dependent, and ZNF334 expression and methylation could be potential prognostic indicators for CRC patients.

### Demethylation effects induced by DAC and AZA mediates ZNF334 upregulation in CRC

**Fig. 2:**
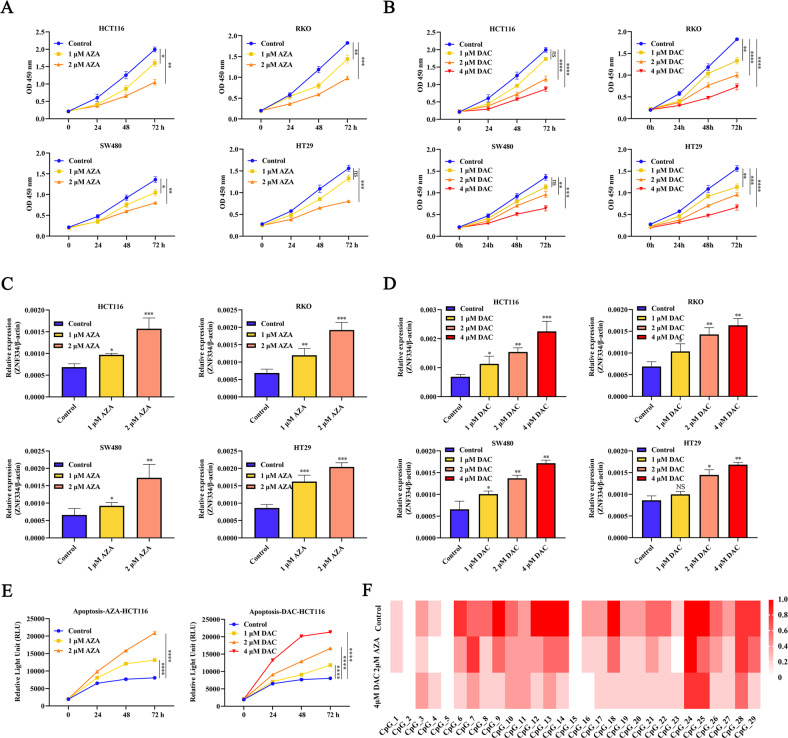
Demethylation of ZNF334 using AZA and DAC in CRC. **A**, **B** A Cell Counting Kit-8 (CCK-8) assay was used to determine the growth of CRC treated with AZA and DAC (*n* = 3). **C**, **D** ZNF334 mRNA expression in CRC after AZA and DAC treatment. Data are shown after normalization to β-actin (*n* = 3). **E** Relative Light Unit was performed to detect apoptotic cells of HCT116 and RKO cells after AZA and DAC treatment (*n* = 3). **F** Genomic DNA was extracted from HCT116 cells treated with AZA and DAC, then subjected to BSP. The promoter methylation of the ZNF334 gene was decreased in CRC cells treated with AZA and DAC after AZA and DAC treatment (*n* = 5). (*p < 0.05; **p < 0.01; ***p < 0.001; ****p < 0.0001, compared with the control group).

To further determine the potential role of DNA methylation in ZNF334 downregulation, two broad-spectrum demethylation agents AZA and DAC were used to treat CRC cells (HCT116, RKO, SW480, and HT29). CCK-8 assay was applied to detect the proliferative ability of the CRC cells. The results showed that the proliferation activity of CRC cells treated with AZA and DAC decreased, and the proliferation activity was inversely correlated with the concentrations of AZA and DAC (Fig. 2A, B). Meanwhile, we observed higher ZNF334 mRNA expression levels after AZA and DAC treatment than in untreated cells (Fig. 2C, D). We further explored whether AZA and DAC could affect CRC cell apoptosis in vitro. The apoptosis of HCT116 was detected by the Relative Light Unit, and results demonstrated that the apoptosis rate of HCT116 cells treated with AZA and DAC was significantly increased than that of the control group (Fig. 2E). BSP product-based sequencing also revealed reduced methylation in the promoter region of the ZNF334 gene in CRC cells treated with AZA and DAC (Fig. 2F). Collectively, these results suggest that the expression of ZNF334 is suppressed by DNA methylation in CRC cells.

### TET1 stimulated the active demethylation of ZNF334

**Fig. 3:**
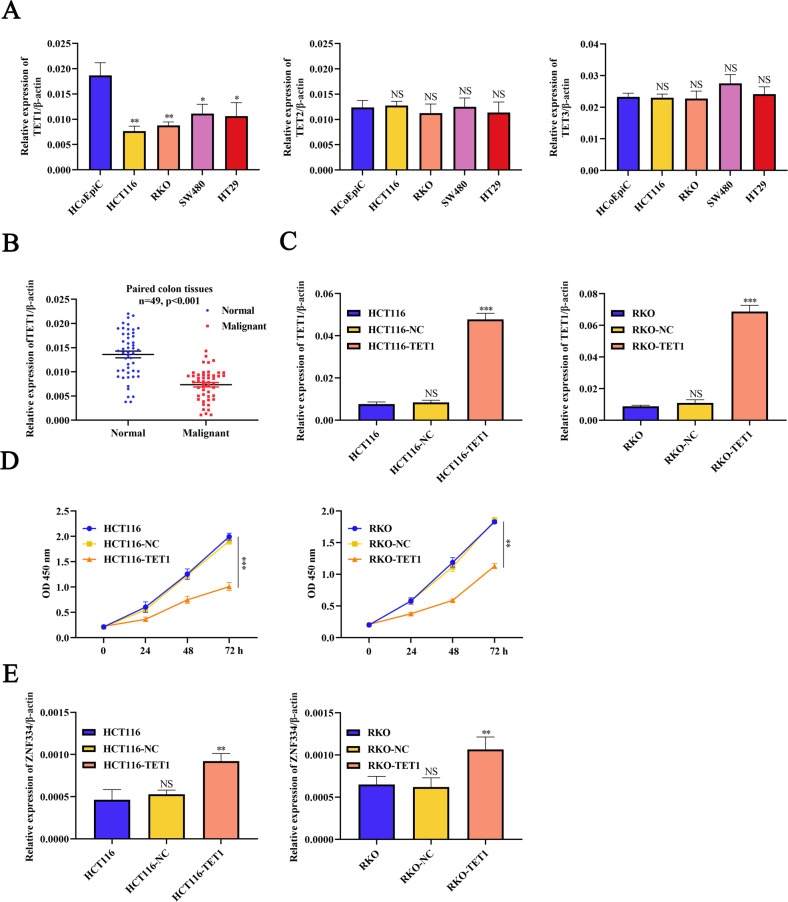
Demethylation of ZNF334 by TET1. **A** The expression of TET1, TET2, and TET3 in HCoEpiC and CRC cells. Data are shown after normalization to β-actin (*n* = 3, *p < 0.05; **p < 0.01, compared with the HCoEpiC group). **B** TET1 mRNA expression in paired CRC tissues (*n* = 49, *p* < 0.001). **C** TET1 mRNA expression in HCT116 and RKO cells after pLVX-MYRF-TET1 infection. Data are shown after normalization to β-actin (*n* = 3, ***p < 0.001, compared with the blank group). **D** A CCK8 assay was performed to determine the growth of HCT116 and RKO cells transfected with pLVX-MYRF-TET1. (*n* = 3, **p < 0.01; ***p < 0.001, compared with the blank group). **E** ZNF334 mRNA expression of HCT116 and RKO cells transfected with pLVX-MYRF-TET1. Data are shown after normalization to β-actin (*n* = 3, **p < 0.01, compared with the blank group). The primer sequences are listed in Supplementary Table 5.

To further investigate the role of methylation in ZNF334 inactivation in CRC cells, we detected the expression of TET (TET1, TET2, and TET3) in HCoEpiC and CRC cells (HCT116, RKO, SW480, and HT29) by Quantitative real-time PCR. The results revealed lower TET1 mRNA expression levels in CRC cells than that in HcoEpiC cells, and the difference has statistical significance. Meanwhile, TET2 and TET3 mRNA expression levels were not significantly different between CRC and HcoEpiC cells (Fig. 3A). Furthermore, Quantitative real-time PCR results demonstrated that TET1 was downregulated in 54.02% of human colorectum tumor tissues, compared with levels in adjacent normal tissues (Fig. 3B, Normal: 0.01357 ± 0.00070 vs. Malignant: 0.00733 ± 0.00046, *p* < 0.0001). Subsequently, HCT116 and RKO cells were selected for further analysis because TET1 expression was lower in these two cells. HCT116 and RKO cells were infected with pLVX-MYRF-TET1. Quantitative real-time PCR analyses suggested that the mRNA expression level of TET1 in HCT116 and RKO cells was significantly higher than that in blank control groups (Fig. 3C). Besides, the assays revealed that TET1 groups dramatically suppressed cell proliferation in HCT116 and RKO cells (Fig. 3D). Finally, Quantitative real-time PCR results displayed that the expression level of ZNF334 was effectively upregulated after overexpression of TET1 in CRC cells (Fig. 3E). Collectively, these results suggest that TET1-mediated DNA methylation is involved in the regulation of ZNF334 expression in CRC cells.

### Targeted demethylation of ZNF334 with dCas9-multiGCN4/scFv-TET1CD-sgRNA system in CRC cells

**Fig. 4:**
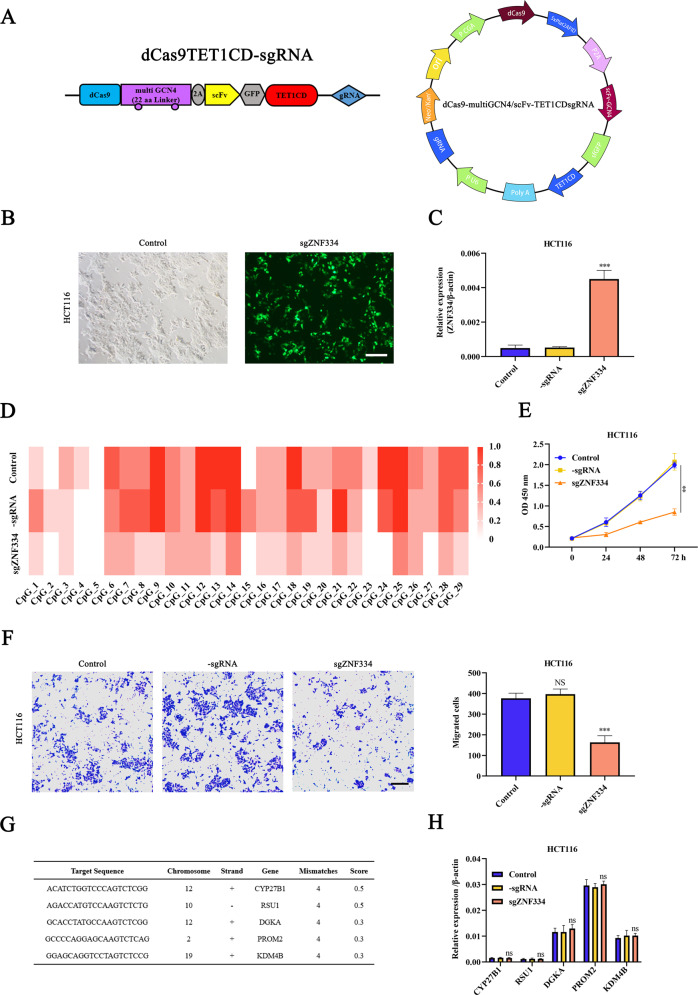
Antitumor effect of the targeted demethylation of ZNF334 in vitro. **A** Schematic description of targeted demethylation via dCas9-multiGCN4/scFv-TET1CD-sgRNA. **B** The fluorescent micrographs show the transfection rate of dCas9-multiGCN4/scFv-TET1CD-sgZNF334 in HCT116 cells. Scale bar = 100 μm. **C** ZNF334 mRNA expression levels were determined by quantitative real-time PCR after transfection (*n* = 3, ***p < 0.001, compared with the sgRNA group). **D** Genomic DNA was extracted from HCT116 cells after transfection, then subjected to BSP. **E** HCT-116 cells transfected with dCas9-multiGCN4/scFv-TET1CD-sgRNA and dCas9-multiGCN4/scFv-TET1CD-sgZNF334 were subjected to the CCK-8 assay (*n* = 3, **p < 0.01, compared with the sgRNA group). **F** The transfected HCT116 cells were collected for a transwell-based migration assay. The migrated cells per frame were counted and analyzed (*n* = 3, Scale bar = 100 μm, ***p < 0.001, compared with the control group). **G** To evaluate the off-target effects of the dCas9-based demethylation system, the top 5 potential off-target sites predicted for sgZNF334 were selected. **H** Quantitative real-time PCR analysis of off-target mRNA expression levels in HCT116 cells transfected with dCas9-multiGCN4/scFv-TET1CD-sgZNF334, compared with those for the blank or sgRNA controls. Data are shown after normalization to β-actin (*n* = 3). The primer sequences are listed in Supplementary Table 5.

To reverse ZNF334 expression by specific demethylation, 5 sgRNAs were designed to construct the dCas9-multiGCN4/scFv-TET1CD-sgRNA-based specific demethylation system, and the targeted sequences of sgRNAs are listed in Supplementary Table 3. We screened the best sgRNA for vector assembly, the sequences of primers used in the assembly cloning are listed in Supplementary Table 4. The system was generated by fusing the catalytic domain of TET1 to the N-terminus of deactivated Cas9 tagged with fluorescent reporter GFP (Fig. 4A). After the transfection of dCas9-multiGCN4/scFv-TET1CD-sgZNF334, the transient transfection efficiency in HCT116 cells reached to 86.48% (Fig. 4B). Quantitative real-time PCR results confirmed the efficient upregulation of ZNF334 at the mRNA levels in HCT116 cells (Fig. 4C). BSP product-based sequencing also demonstrated reductions in CpG methylation in dCas9-multiGCN4/scFv-TET1CD-sgZNF334-transfected CRC cells (Fig. 4D). Subsequently, the proliferation rate of HCT116 cells was measured using the CCK-8 method. Assays revealed that dCas9-multiGCN4/scFv-TET1CD-sgZNF334 dramatically suppressed the proliferation of HCT116 cells (Fig. 4E). Furthermore, fewer migrated HCT116 cells were observed in the dCas9-multiGCN4/scFv-TET1CD-sgZNF334-transfected groups than in the control groups (Fig. 4F). These results demonstrated the anti-tumor effect of the targeted demethylation of ZNF334 in CRC. Further, identifying off-target cleavage by any Cas9-based technique in a genome of interest is important, especially in treating all kinds of cancer. Thus, we used the COSMID online tool (https://crispr.bme.gatech.deu/) [29] to select the top 5 potential off-target loci to examine whether their mRNA transcription levels were altered due to off-target demethylation by dCas9-multiGCN4/scFv-TET1CD-sgZNF334 (Fig. 4G). As shown in Fig. 4H, no significant differences in the mRNA expression levels of these potential off-target genes were detected between the dCas9-multiGCN4/scFv-TET1CD-sgZNF334 group and dCas9-multiGCN4/scFv-TET1CD-sgRNA or blank control group (Fig. 4H). These results suggested that the dCas9-multiGCN4/scFv-TET1CD-sgZNF334 was effective and specific for ZNF334 demethylation.

### Targeted demethylation of ZNF334 inhibits colorectal cancer growth

**Fig. 5:**
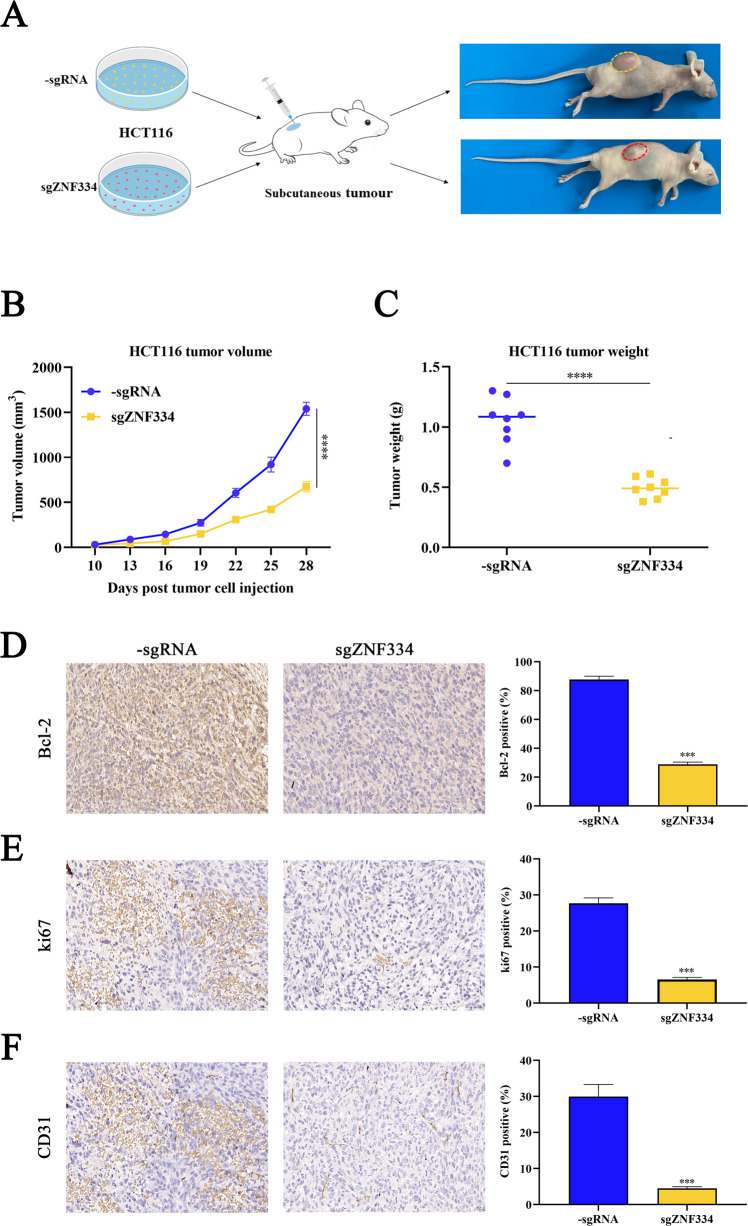
The targeted demethylation of ZNF334 suppresses HCT116 cell tumor growth in CRC xenograft mice. **A** The diagram of the antitumor effect of the targeted demethylation of ZNF334 in vivo. **B**, **C** Tumor volume and end-stage tumor weight after the injection of HCT116 cells stably transfected with dCas9-multiGCN4/scFv-TET1CD-sgRNA and dCas9-multiGCN4/scFv-TET1CDsgZNF334 (*n* = 8, ****p < 0.0001, compared with the sgRNA group). **D**–**F** Immunohistochemical staining assay detected the apoptosis and proliferation in the tumor tissues, and the percentages of Bcl-2, Ki67, and CD31-positive cells were analyzed (*n* = 3, Scale bar = 20 μm, ***P < 0.001, compared with the sgRNA group).

Next, to evaluate the role of the targeted demethylation of ZNF334 on tumor growth in vivo, 5 × 10^6^ HCT116 cells transfected with the dCas9-multiGCN4/scFv-TET1CD-sgZNF334 or the dCas9-multiGCN4/scFv-TET1CD-sgRNA were injected into the flank of female wild-type (WT) BALB/c nude mice to establish a subcutaneous tumor model (Fig. 5A). Our results indicated that the targeted demethylation of ZNF334 significantly reduced the tumor volume of HCT116 cells (Fig. 5B, tumor volume: sgRNA: 514.7 ± 208.8 mm^3^ versus sgZNF334: 242.6 ± 90.8 mm^3^). Targeted demethylation of ZNF334 also dramatically inhibited the weight of HCT116 cells (Fig. 5C, tumor weight: sgRNA: 1.053 ± 0.069 g versus sgZNF334: 0.495 ± 0.029 g). Additionally, more apoptotic and fewer proliferative cells were observed in HCT116 tumors after the targeted demethylation of ZNF334 (Fig. 5D, E). We also determined the effect of the targeted demethylation of ZNF334 on tumor vascularization by staining for endothelial-specific antigen CD31. As shown in Fig. 5E, the treatment with the targeted demethylation of ZNF334 leads to a significant reduction in CD31 staining (Fig. 5F). Collectively, these data imply that the targeted demethylation of ZNF334 is a potential anti-tumor strategy for the treatment of CRC.

## Discussion

The ZNFs are one of the largest families of transcription factors, in which the zinc-finger domain can bind to gene promoter regions and play an effective role in regulating cell proliferation, differentiation, and apoptosis [10, 30]. Accumulating evidence suggests that ZNFs play an important role in many important biological processes related to tumorigenesis [31]. ZNF334 is a new describe member of the KRAB-ZNFs family, which has been shown to be associated with RA, TNBC, and HCC [19, 25–28]. However, the expression of ZNF334 in CRC and its regulatory effect on tumor proliferation and apoptosis remain unclear. In this study, we found that ZNF334 was downregulated in CRC by analyzing the TCGA data, CRC cells, and clinical tissues.

Previous studies have shown that epigenetic changes in the genome are strongly associated with the occurrence of cancer. DNA hypermethylation of gene promoters and other gene regulatory regions has been frequently reported in conjunction with silenced/downregulated gene expression [32, 33]. The downregulation of many major tumor suppressor genes is associated with methylation-mediated transcription silencing. After induction by demethylation, the expression of tumor suppressor genes in these regions increases [34, 35]. Thus, DNA methylation has been studied as an epigenetic biomarker of different types of cancer. In this experiment, we observed that the methylation rate of CpG sites in the promoter region of the ZNF334 gene decreased and the expression of ZNF334 increased after the treatment of CRC cells with the Broad-spectrum demethylation agents DAC and AZA. Meanwhile, a strong negative correlation between ZNF334 expression and ZNF334 methylation was detected in CRC patients, confirming that downregulation of ZNF334 expression was mediated by hypermethylation of the ZNF334 promoter.

The diminished expression of TET proteins and loss of 5-hydroxymethylcytosine (5hmC) in many tumors suggests a critical role in the maintenance of this epigenetic modification. The family of TETs consists of TET1, TET2, and TET3. The methylcytosine dioxygenase TET1 is an important regulator of 5hmC in embryonic stem cells. Here, we demonstrate that TET1 is involved in the regulation of ZNF334 methylation in CRC cells. Hence, a dCas9-multiGCN4/scFv-TET1CD-sgZNF334 demethylation system was constructed to reverse the expression of ZNF334. We assessed dCas9-multiGCN4/scFv-TET1CD-sgZNF334 was effective and specific for targeted DNA demethylation. Meanwhile, we demonstrated the anti-tumor effects of dCas9-multiGCN4/scFv-TET1CD-sgZNF334 in CRC in vitro and in vivo.

In conclusion, our study demonstrated that high DNA methylation levels in the promoter region could down-regulate the expression of ZNF334 and participate in the occurrence of CRC. Next, we used dCas9-multiGCN4/scFv-TET1CD-sgZNF334 to specifically down-regulate the methylation level of the ZNF334 promoter region, which was shown to restore ZNF334 expression and to have significant antitumor effects both in vitro and in vivo. In general, we explored the biological role of tumor suppressor gene ZNF334 in the occurrence of CRC and provided a new target and clinical evidence for the early diagnosis and precise treatment of CRC.

## Methods

### Cell and antibodies

The CRC cell lines (HCT116, SW480, RKO and HT29) HcoEpiC were bought from American Type Culture Collection (ATCC, Manassas, VA, USA) and cultured in DMEM (Invitrogen, MA, USA) containing 10% fetal bovine serum (FBS) and antibiotics.

The following antibodies were utilized in this study: CD31 (ab281583, Abcam, London, UK), Bcl-2 (AF6139, Affinity Biosciences), Ki67 (ab16667, Abcam, London, UK).

### Animals

The Model Animal Research Center of Nanjing University (Nanjing, China) sold us five-week-old female BALB/c nude mice, and we kept them in a controlled environment (20–22 °C, 12 h light and 12 h dark cycles, and 45–55% relative humidity). For each experiment, the mice were randomly divided into three to five per cage, the ears were marked, and then recorded blindedly (without group information). All mice acclimatized to the environment for one week and were provided with a casual diet and pure water. A subcutaneous cancer model was established by injecting 5 × 10^6^ sgZNF334 and sgRNA HCT116 cells into the flanks of female BALB/c nude mice. Tumor size was determined by length and width measurements. Tumors of each mouse were collected and weighed for histopathology studies. All animal studies were performed in accordance with US National Institutes of Health guidelines for the care and use of Laboratory Animals and approved by the Southwest Minzu University Animal Care and Use Committee.

### Tissue sample collection and processing

Frozen samples from 93 patients with CRC were obtained from the Biological Specimen Banks (West China Hospital, Sichuan University, China). All these patients were diagnosed with CRC by two independent pathologists. Of the 93 cases, 49 had tumors with paired adjacent normal tissues. The clinicopathological parameters and follow-up data, including age at diagnosis, sex, tumor size, National Institutes of Health risk classifications, survival, and distant metastasis, were extracted from the patients’ medical records. The study protocol was approved by the Research Ethics Board of West China Hospital, Sichuan University, China. Written informed consent was obtained from each patient.

### Construction of targeted demethylation vector

This study used the all-in-one vector (Addgene plasmid 82559) that includes a network of Cas9 peptides (linker length: 22aa), the sfGFP-TET1CD antibody, and the gRNA expression system. By linearizing the Addgene plasmid 82559 AflII sites, including the gRNA insert segment by Gibson assembly, cloning was accomplished. To make the volume to 10 μl, combined the plasmid 82559 digested with AflII (50 ng). Incubate for 15 minutes at 50 °C. Use competent Escherichia coli and the 5 μl reaction mixture given above to perform bacterial transformation. Then, distributed the competent Escherichia coli on a solid medium containing kanamycin to identify the transformed clones that have succeeded. Purify the plasma and cultivate the colony. The target sequence was described in the supplementary file.

### Cell transfection

Seed the cells into 12-well plates (Corning) according to the product instructions. Then, transfected plasmids into cells using DNA Transfection Reagent. 4 days following the transaction, cells were collected. The cells received extended G418 therapy to create cell lines that systematically expressed the demethylation system. RNA and genomic DNA had been extracted from selected colonies to study.

### DNA methylation assay

Following the manufacturer’s instructions, genomic DNA was isolated from cells and tissues using the Tissues and Cell Genomic DNA Purification Kit (DP021, GeneMark), the CpGenome Turbine Bisulfite Modification Kit (Merck KGaA) was then used for modification. The modified DNA was amplified using the PCR primers listed in supplemental Table 1. Bisulfite sequencing analysis was done to conduct a methylation analysis of the neighborhood. In brief, at least five clones were sequenced after the amplified fragment was ligated to the TOPO vector (TsingKe). Methylation analysis tools were used to examine these sequences (QUMA). In addition to studying this quick and effective technique for possible clinical application, we performed pyrosequencing analysis (Oebiotech) to quantify measure the methylation level of tissue samples and relate it to the overall survival rate of CRC patients. After the target sequences was converted by bisulfite, two CpG sites were studied by pyrosequencing. The 3′-end of the sequencing primer was the place where the pyrosequencing reaction began. Each sample well received one nucleotide (A, T, C, and G) at a time. Every time a base that was compatible with the base in the PCR product was introduced, it was incorporated into the expanding DNA strand, triggering an enzymatic cascade and the creation of light. At every dispensation, to measure light intensity and visually displayed in a program. Data on methylation was displayed as a percentage of the overall average methylation of all CpG sites.

### Real-time PCR

Total RNA was extracted from the sample using Trizol reagent (Invitrogen, Carlsbad, CA, USA). Following that, RT-PCR was performed on RNA samples (1 μg) using the TaKaRa One-step RT-PCR kit. Electrophoresis separation of all PCR products with a 1% agarose gels. The resultant complementary DNA for RT-PCR was examined in triplicate with SYBR Green. In Supplementary Table 4, the primer sequences for quantitative RT-PCR (qRT-PCR) were displayed.

### Cell proliferation assay

Cells from 96 well plates were planted (2000 cells/well) and kept alive. The proliferation of cells after adhesion was determined. According to the product instructions that 10 μl of the Dojindo cell counting kit-8 was used and incubated for 2 h. Fluorescence measurements (450 nm) were taken in the microplate reader.

### Relative Light Unit assay

Cells were seeded 5000 cells per well in DMEM culture medium in white 96-well plates with clear bottom in triplicate per experimental condition. Relative Light Unit was measured at 0, 24, 48, and 72 h after cell seeding.

### Transwell migration assay

Transwell chambers (Corning) had a membrane with an 8 μm hole in the bottom. Matrigel (Sigma) was applied to the chambers to test their invasive ability. For the migration test, no matrigel was applied. The bottom layer was filled with 10% DMEM culture solution containing fetal bovine serum. The top layer of the Transwell chambers had a 200 μl volume. 5 × 10^5^ CRC cells were used as the inoculum. Remove the wells and preserved in a solution made of methanol and glacial acetic acid (3:1) for 30 min after the cells had been cultivated for 48 h at 37 °C and 5% CO^2^. Next, dyed with 0.1% crystal violet. After staining, observing and taking pictures under an electron microscope.

### Immunohistochemical (IHC) staining

The 4 µM sections of paraffin-embedded tissues were cut, baked for two hours at 58 °C, dewaxed with xylene for 30 min, submerged in a gradient of ethanol for five minutes each, and then rinsed for five minutes with distilled water. After adding the sodium citrate antigen repair solution, the cells were boiled in boiling water for 20 min, and then washed three times for five minutes each with PBS solution. The sections were then incubated with primary antibodies CD31, Ki67, and Bcl-2 in a refrigerator at 4 degrees celsius for 12 h. After removal, the product was reheated for an hour while the film was cleaned three times for five minutes each with PBS solution. After one minute of color development with diaminobenzidine (1:50), the reaction was stopped using distilled water. Hematoxylin was added, hydrochloric acid ethanol was differentiated, then the sections were dewatered with gradient ethanol and rinsed 3 times with xylene to translucency. After sealing the slices with neutral glue, observing and taken pictures under an electron microscope. As indicated by CD31, Ki67, and Bcl-2 staining in tumors, the number of cancer cells expressing the proteins was counted, and the correlation was examined.

### Statistical analyses

All experiments were repeated three to five times. Quantitative variables are presented as mean ± standard deviation (SD) and were analyzed using the Student’s *t* test or one-way analysis of variance. Categorical variables are expressed as percentages and statistical significance was tested using the Chi-squared or Fisher’s exact tests. Kaplan–Meier curves and the log-rank test were used to compare survival times among groups. Symbols used to denote significance are as follows: *P < 0.05, **P < 0.01, ***P < 0.001, ****P < 0.0001 and ns (no statistical significance). The relevant numbers make a clear statement about the number of duplicated experiments. “n” represents the number of mice and tissue used in our experiments. All statistical calculations in this study were carried out using GraphPad Prism 9.0 software.

## Supplementary information

Targeted DNA demethylation of the ZNF334 promoter inhibits colorectal cancer growth

The reproducibility checklist

## Data Availability

All datasets generated and analyzed during this study are included in this published article and its Supplementary Information files. Additional data are available from the corresponding author on reasonable request.
