# Supplementary material for: Targeted DNA demethylation of the ZNF334 promoter inhibits colorectal cancer growth
Source: Cell Death Dis. 2023 Mar 25;14(3):210. doi: 10.1038/s41419-023-05743-x (PMC10039945; doi:10.1038/s41419-023-05743-x)
Supplement: Supplementary file 1 — Targeted DNA demethylation of the ZNF334 promoter inhibits colorectal cancer growth [file 41419_2023_5743_MOESM1_ESM.docx]

**Supporting Information**

**Targeted DNA demethylation of the ZNF334 promoter inhibits colorectal cancer growth**

Bin Yang^1^, Haiyu Tang^1^, Nan Wang^1^, Jian Gu^1^, Qin Wang^1,2,^*

^1^School of Pharmacy, Southwest Minzu University, Chengdu, Sichuan 610225, China.

^2^BMI Center for Biomass Materials and Nanointerfaces, College of Biomass Science and Engineering, Sichuan University, Chengdu, Sichuan 610065, China.

*Email: jiajiawangqin@163.com

**Supplementary Tables**

**Supplementary Table 1**

| **Characteristics** | **Number of patients** |
| --- | --- |
| **Gender(%)** |  |
| Male | 48(51.6) |
| Female | 45(48.4) |
| **Age(%)** |  |
| ≤60 | 52(55.9) |
| >60 | 41(44.1) |
| **Dukes stage(%)** |  |
| I | 3(3.2) |
| II | 49(52.7) |
| III | 35(37.6) |
| IV | 6(6.5) |
| **Differentiation** |  |
| G1* | 34（36.6） |
| G2 | 56（60.2） |
| G3** | 3（3.2） |
| **Tumor diameter（%）** |  |
| ≤5cm | 37（39.8） |
| ＞5cm | 56（60.2） |
| **Tumor Site** |  |
| Distal | 34（36.6） |
| Proximal | 59（63.4） |
| **Lymph vascular invasion** |  |
| Yes | 5（5.4） |
| No | 88（94.6） |
| **Perineural invasion** |  |
| Yes | 1（1.1） |
| No | 92（98.9） |

**Supplementary Table 2**

**Sequences of primers used in the bisulfite DNA sequencing.**

| Target gene | Forward primer (5’-3’) | Reverse primer (5’-3’) |
| --- | --- | --- |
| ZNF334 | GATTATAGGAAATTTTTTTTTTAAAGATAGGG | AACTCCTCAAAAATCCCTC |

**Supplementary Table 3**

**The targeted sequences of dCas9-based demethylation vector.**

|  | The guide sequences |
| --- | --- |
| sgZNF334-1  sgZNF334-2  sgZNF334-3  sgZNF334-4  sgZNF334-5 | CGGGCTGCAGGACCCTCACC  TCAGGAGCCCAATCCTGCCC  ACCAGGGATTCACGGGCTGC  CGGCGGGACCAGGGATTCAC  TCGGCGGGACCAGGGATTCA |

**Supplementary Table 4**

**Sequences of primers used in the assembly cloning.**

|  | Sequences |
| --- | --- |
| Forward primer (5’-3’)  Reverse primer (5’-3’) | ATTTCTTGGCTTTATATATCTTGTGGAAAGGACGAAACA  CGGACTAGCCTTATTTTAACTTGCTATTTCTAGCTCTAAAAC |

**Supplementary Table 5**

**S Sequences of the primers used in qRT-PCR assay.**

| Target gene | Forward primer (5’-3’) | Reverse primer (5’-3’) |
| --- | --- | --- |
| β-actin | GAGAAAATCTGGCACCACACC | GGATAGCACAGCCTGGATAGCAA |
| ZNF334 | AGGGGAGACAGACTGAAAGGA | GTGAGGCTTGTCTTCACACG |
| TET1 | GATGACAGAGGTTCTTGCACAT | AGGTTGCACGGTCTCAGTGT |
| TET2 | ATACCCTGTATGAAGGGAAGCC | CTTACCCCGAAGTTACGTCTTTC |
| TET3 | CGATTGCGTCGAACAAATAG | CTCCTTCCCCGTGTAGATGA |
| CYP27B1 | AAGTTCCGGTTTTGCCCATC | ACACAGCGGACACTTGGATA |
| RSU1 | GGATGTCAACGGCCTCTTT | TGGCACCATTGTTAGCTTGT |
| DGKA | CTCAATAGCGCCATCGAAGC | CGACGATAATGGCAATCAGCAC |
| PROM2 | AGGTCCAGGCTCTGTGTGTC | GCTCAACGACTCCTACGACC |
| KDM4B | CTTCACGCAGTACAATATCC | CGTCGTCATCATACAAAGAG |
